# Analysis of dietary interventions. A simple payoff matrix for display of comparative dietary trials

**DOI:** 10.1186/1475-2891-7-24

**Published:** 2008-08-29

**Authors:** Richard D Feinman, Eugene J Fine, Jeff S Volek

**Affiliations:** 1 Department of Biochemistry, State University of New York Downstate Medical Center, Brooklyn, New York, USA; 2 Department of Nuclear Medicine, Albert Einstein College of Medicine, Bronx, New York, USA; 3 Department of Kinesiology, University of Connecticut, Storrs, Connecticut, USA

## Abstract

**Objective:**

To provide a simple method for presentation of data in comparative dietary trials.

**Methods:**

Individual data from each diet are ranked and all possible paired comparisons are made and displayed in a pay-off matrix which can be color-coded according to the magnitude of the differences between the two diets. Probability of outcome can be calculated from the fraction of matrix elements corresponding to specified conditions. The method has the advantage of emphasizing differences and providing the maximum amount of information.

**Results:**

The method was tested with values from the literature and allows intuitive sense of the comparative effectiveness of the two diets. In a test case in which a cross-over study had been performed the matrix derived from theoretical paired comparisons (treating the data as two parallel studies) was consistent with the results from the actual pairing in the cross-over.

**Conclusion:**

The matrix method is a simple way of providing access to the differences between dietary trials. It exaggerates differences but can be used in combination with group statistics that, conversely, provide reliability at the expense of detailed information.

## Background

It is increasingly recognized that individual variation is an important consideration in dietary interventions for weight loss, cardiovascular disease and other conditions. Position papers of scientific organizations make the point that it is "unlikely that a single strategy is best for all" (e.g. [1]). It seems, however, that the principle is more widely applied in the breach than in the observance and most dietary trials continue to report means and standard deviations or other measures of group statistics. The use of group statistics in a dietary intervention implies not only that the population is uniform but, in addition, that there is a best diet for all individuals. In fact, given the difficulty in effecting weight loss, the test of a dietary strategy might better be whether it can be made to work for anybody. In this sense, the outliers become the key parameters in diet and the probability of reaching some criterion, rather than average behavior is intuitively a useful measure of efficacy of a diet. Further, weight loss involves inconvenience and dedication and a prospective dieter or physician is sensibly asking about relative payoffs of different dietary approaches – in essence, which diet to bet on. Thus, what one wants from a dietary comparison is to be able to compare individual performances on different diets rather than group values. Non-parametric statistics based on ranking such as the Mann-Whitney tests are applicable but because the rank becomes the primary variable, information is lost that a potential dieter would like to know.

Here we describe a simple way to present individual data in comparative dietary trials that preserves the maximum amount of information and allows judgments of efficacy to be made. The idea is based on assessment of all possible paired comparisons and deduction of benefit from evaluation of a pay-off matrix, analogous to a matrix comparing different outcomes in games of strategy [2].

The experimental ideal for taking account of individual variation is the cross-over protocol, that is, paired variables, in which subjects alternate between the two different diets. In this case, an odds-ratio for specified differential performance or some similar parameter will give the information that a dieter might want. Because it is difficult to perform any dietary intervention, however, a cross-over experiment is not always feasible and one may have to compare two groups in parallel who cannot generally be assumed to have uniform responses to diet.

## Methods

Individual results for the two diets to be compared are ranked according to outcome, for example, weight loss. All possible differences are calculated and these differences constitute the elements of a payoff matrix. The matrix elements can be color coded to indicate particular levels of relative payoff, e.g. > 2 kg difference in weight loss. Probabilities of particular outcomes can also be calculated. The underlying rationale is that, since we don't know which subject in intervention A should be matched with which subject in intervention B, we consider all such possibilities and consider all the outcomes. The method has the advantage that it does not assume any particular distribution. It has the disadvantage that it assumes that the sample distribution of responses is somehow representative of the population distribution, and it therefore tends to over-emphasize differences.

The matrix presentation brings out the qualitative features of individual responses and allows a graphic representation of the comparative effects. It emphasizes differences and therefore is exaggerated in comparison with sample means which tend to emphasize consistency.

We illustrate the method with data from a paper by Volek, et al. [3]. In this cross-over study, 15 men and 13 men were assigned to a low fat (LF) diet or a very low carbohydrate ketogenic diet (VLCKD). After a certain period subjects were switched to the other diet. We first treat the data as if this were a parallel experiment, that is, as if the LF and the VLCKD group were separate individuals. We then ask how the results compare to the actual outcomes where the pairings are known.

**Figure 1:**
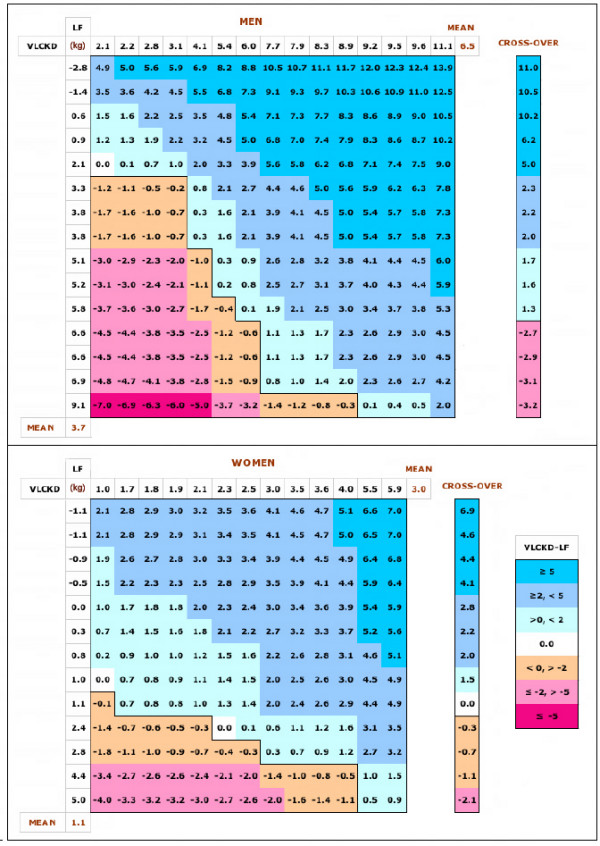
**Payoff matrix for dietary comparisons**. Matrices show (theoretical) paired comparisons: Weight loss (in kg) for each individual in the VLCKD is shown in rank order across the top of the matrix (X-axis). Weight loss for the LF is shown down the side of the matrix (X-axis). Each matrix element shows the difference between the value for the VLCKD (column) and the value for the LF (row):VLCKD-LF. Positive values indicate more weight loss for the VLCKD value than the LF, negative values indicate the reverse. Data are from reference [3] in which subjects were assigned to two diets with roughly similar caloric levels (VLCKD: 1855 kcal/d; LF: 1550 kcal/d) differing in nutrient composition: VLCKD = %carbohydrate:fat:protein = ~9:63:28, LF, ~58:22:20. After a fixed period (50 days for men; 30 days for women) subjects switched to the other diet. Data in the matrices are for performance in each phase. In the cross-over data, weight loss for each subject from the LF phase is subtracted from weight loss in the VLCKD phase (regardless of which came first in the experiment) and displayed in rank order.  Color-coding as indicated in the figure.

The matrix shown in Figure 1 represents the differences in all responses for the two diets (regardless of order). The horizontal row across the top of the matrix shows the individual values for weight loss on the VLCKD, while the vertical column on the left shows individual weight losses on LF. The matrix elements are the differences between the two diets, that is, the column value minus the row value. Positive values indicate that the VLCKD did better than the low fat. Examination of the color coding of the matrix shows that, consistent with the mean responses, there is a clear choice of the VLCKD. The actual probability predicted by the theoretical pairing shown in Table 1 are calculated from the total number of matrix elements for each condition divided by the total.

**Table 1:** Probability of differences in outcomes in diet comparisons

		***PROBABILITY***	
		MATRIX	X-OVER
			
***MEN***	**VLCKD > LF**	**0.73**	**0.73**
	LF > VLCKD	0.27	0.27
	**VLCKD – LF > 2**	**0.59**	**0.53**
	LF – VLCKD > 2	0.17	0.27
	**VLCKD – LF > 5**	**0.31**	**0.33**
	LF – VLCKD > 5	0.02	0
			
***WOMEN***	**VLCKD > LF**	**0.78**	**0.62**
	rLF > VLCKD	0.21	0.31
	**VLCKD – LF > 2**	**0.69**	**0.54**
	LF – VLCKD > 2	0.09	0.31
	**VLCKD – LF > 5**	**0.09**	**0.31**
	LF – VLCD > 5	0	0

The number of matrix elements corresponding to each condition are divided by the total number of matrix elements (paired differences) in the matrices in Figure 1. For cross-over, difference for results (VLCKD – LF) for each subject are divided by the number of subjects.

The matrix display shows that, beyond average performance, those subjects who benefited more from carbohydrate restriction compared to reduced fat generally did so in a big way (> 5 kg differential weight loss). Table 1 summarizes the probabilities of the various theoretical outcomes. An individual dieter is not guaranteed better outcome on either diet but the table demonstrates that they have a far better chance of showing dramatic results if they go with the VLCKD.

Volek's experiment [3] had a second phase in which subjects switched diets allowing a test of the matrix method, that is, one can ask how the actual within-subject comparisons compare to the anticipated outcomes of the matrix presentation. Table 1 compares the probabilities from the matrix analysis and from the data in the cross-over experiment. The table shows that there is generally good agreement – fortuitously good for the overall comparison of the two groups – and it is clear that the matrix predicts, and only slightly exaggerates, the value of the VLCKD compared to LF.

## Discussion

Group statistics tend to destroy information in order to gain reliability. The case might be made that not every experiment should have the same standards for the relative importance of these two parameters. In experiments where people can reasonably be expected to have similar responses, such as drug trials, an expectation value based on average outcome is the most relevant. On the other hand, given the difficulty of staying on any diet, the high non-Gaussian prevalence of obesity in the general populations [4] and the variability due to hidden variables (like non-exercise activity thermogenesis (NEAT, [5]), the prospective dieter is really asking which diet to bet on, that is, what is the best possible payoff for each diet and what are the odds, analogous to picking a strategy in the theory of games [2]. In the end, nobody loses an average amount of weight and the frequently quoted conclusion that low-carbohydrate and LF diets are the same at one year (e.g., [6]) might be further enlightened by individual analysis along the lines suggested from a matrix presentation.

Group statistics are integrative methods and decrease the signal to noise ratio and increase reliability but, because they obscure differences can repress future experiments. The matrix method is a derivative method and therefore increases the noise. It has reduced reliability but because it highlights potential differences, can provide a guide to future experiment and is hypothesis generating. Outlier data can be compared against prospective trials using Chi-square or Fischer exact testing to improve the reliability of the original observation. Use of both methods would provide maximum information about the nature of the comparison. Finally, while the example given is a weight loss experiment, the method is generalizable to all experimental parameters, such as lipid profile, that may be under the control of diet.

Comparison of matrix data from the results of multiple experiments will be required to make the most reliable conclusions. Along these lines, a comparison of the numerical differences between LF and low carbohydrate diets, regardless of statistical significance showed the likelihood that the carbohydrate-restricted diet was better [7], that is, treated as separate Bernoulli trials, the results were not likely to be random. The matrix method would demonstrate whether the numerical benefit of the low carbohydrate diet was due to a sub-group with unusually good performance.

## Competing interests

The authors declare that they have no competing interests.

## Authors' contributions

Authors contributed equally to the manuscript.
